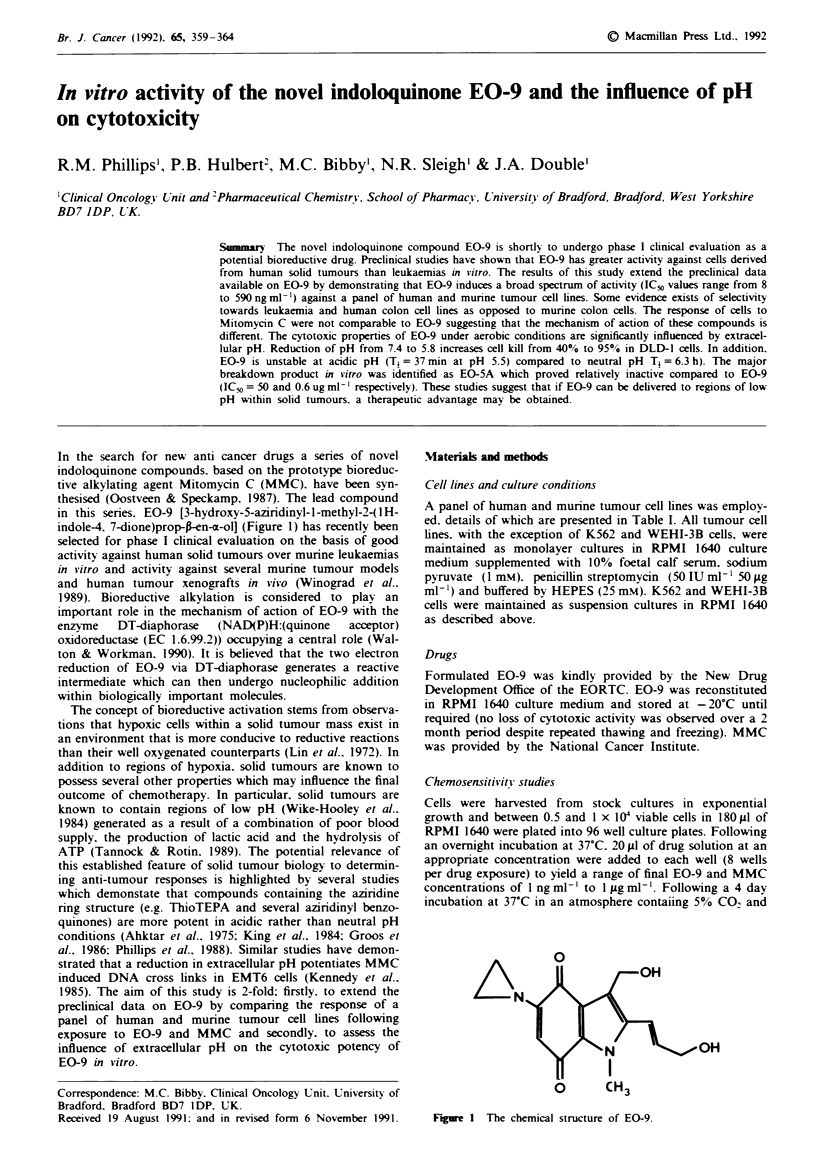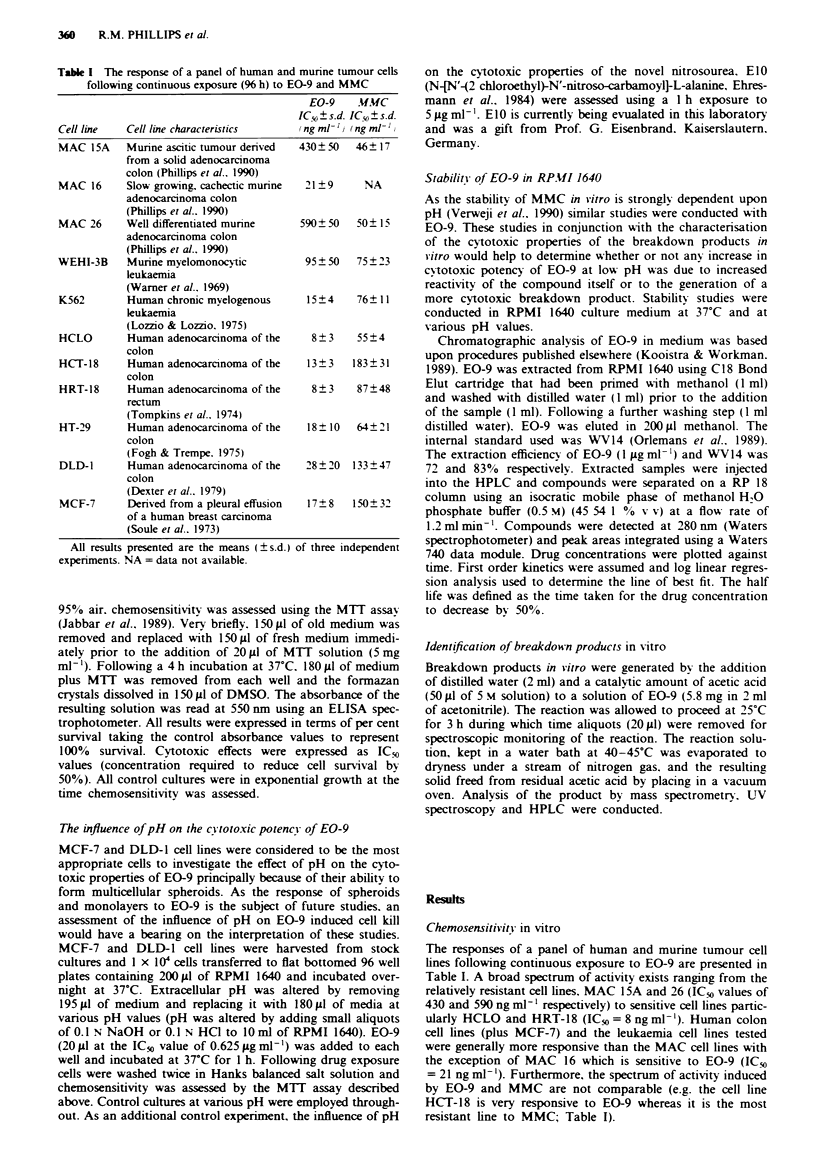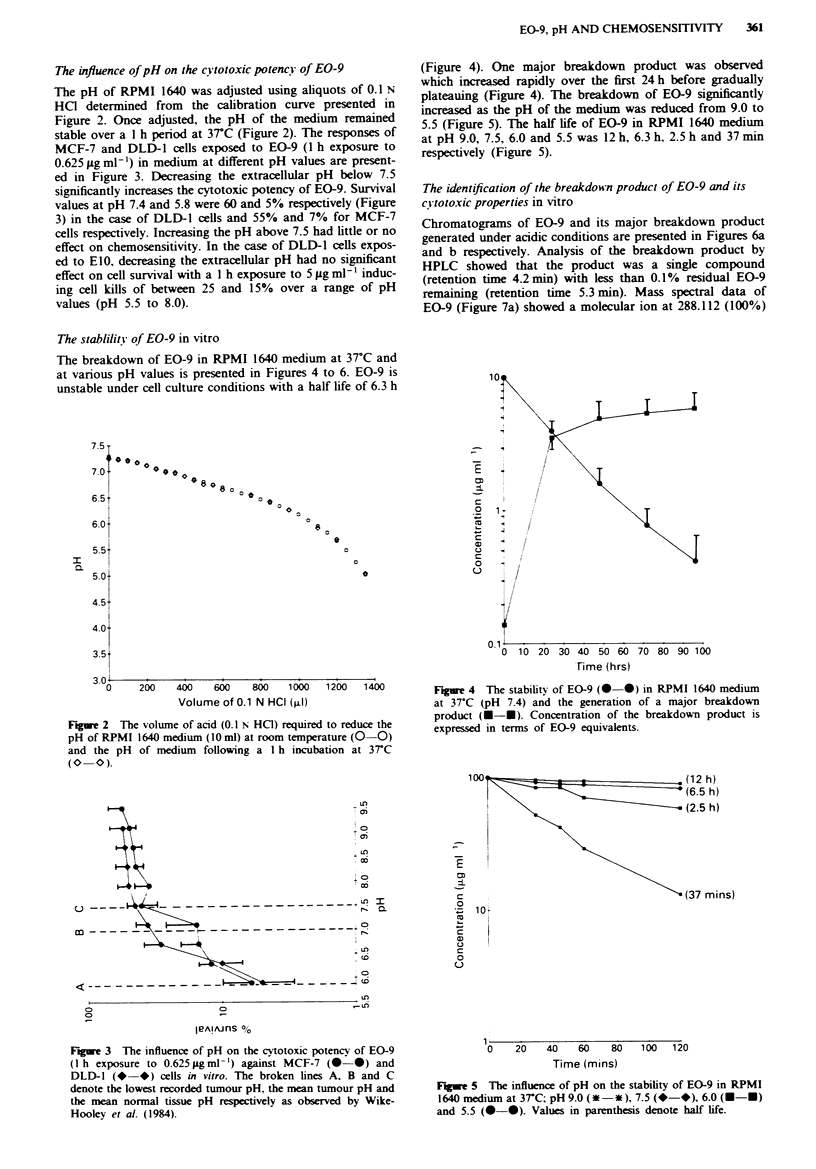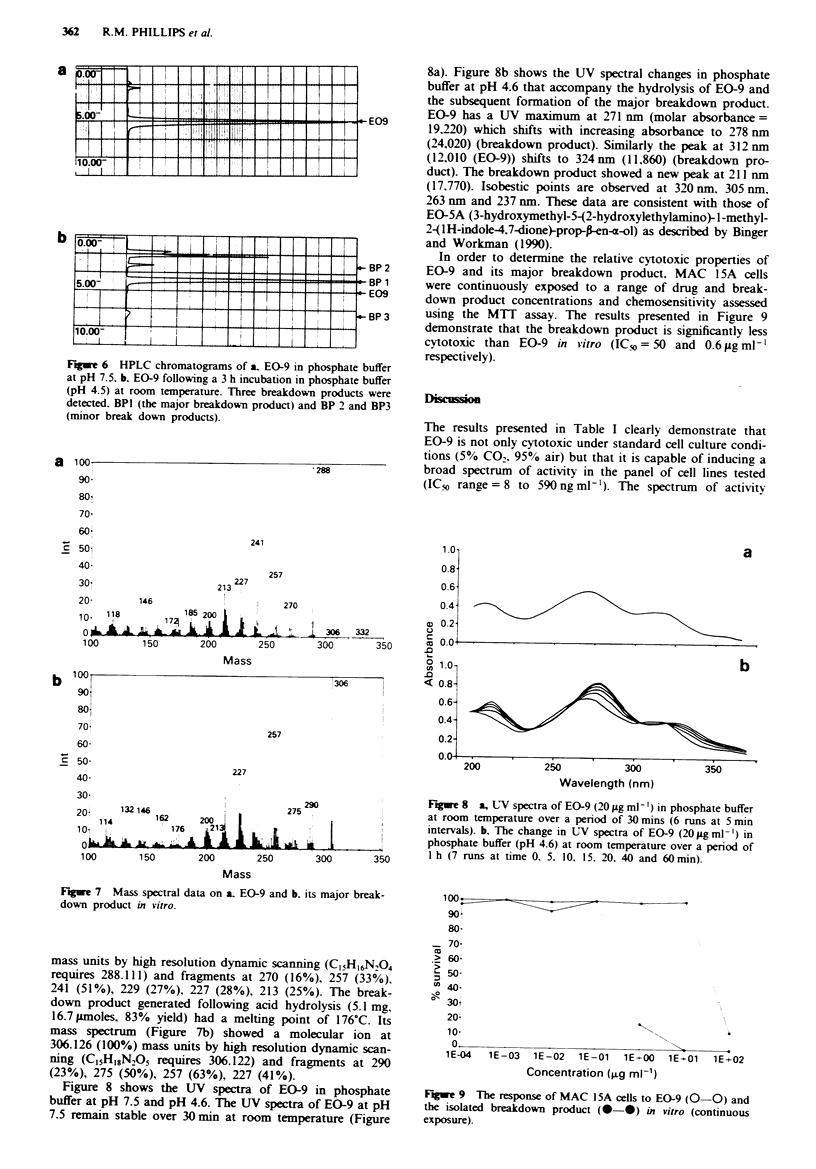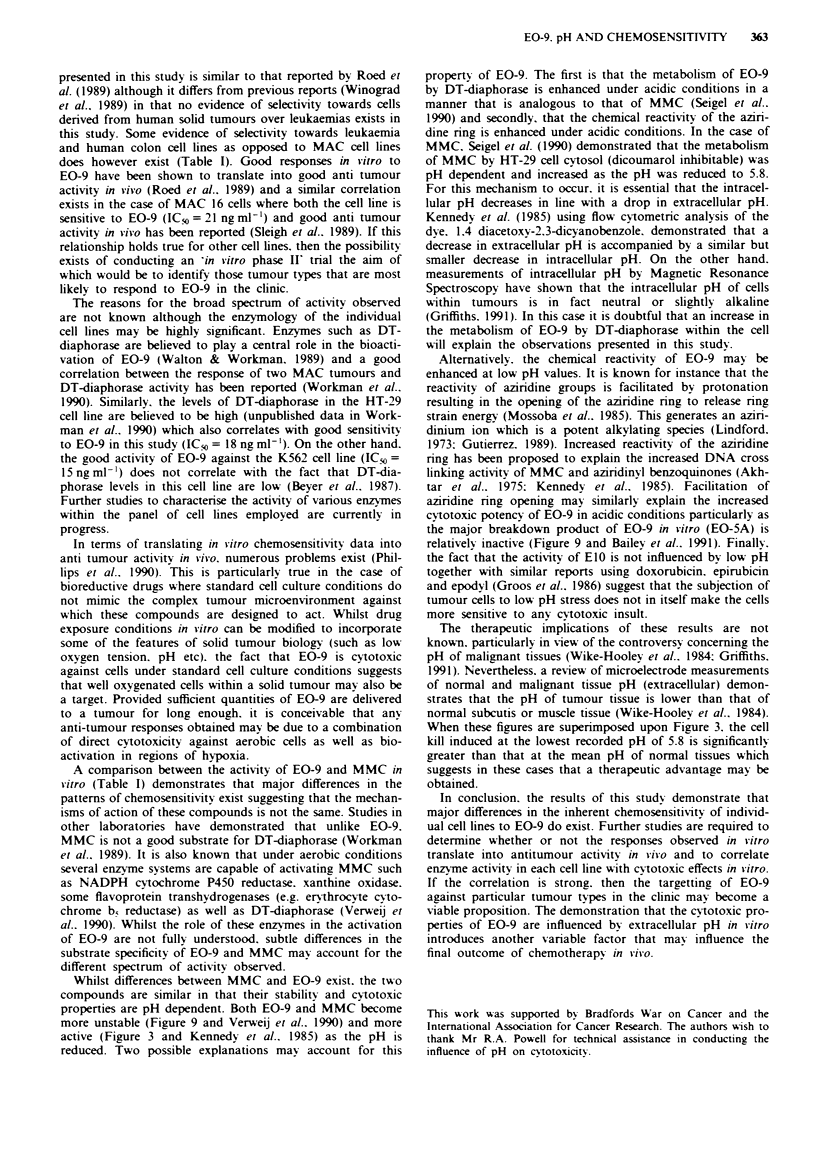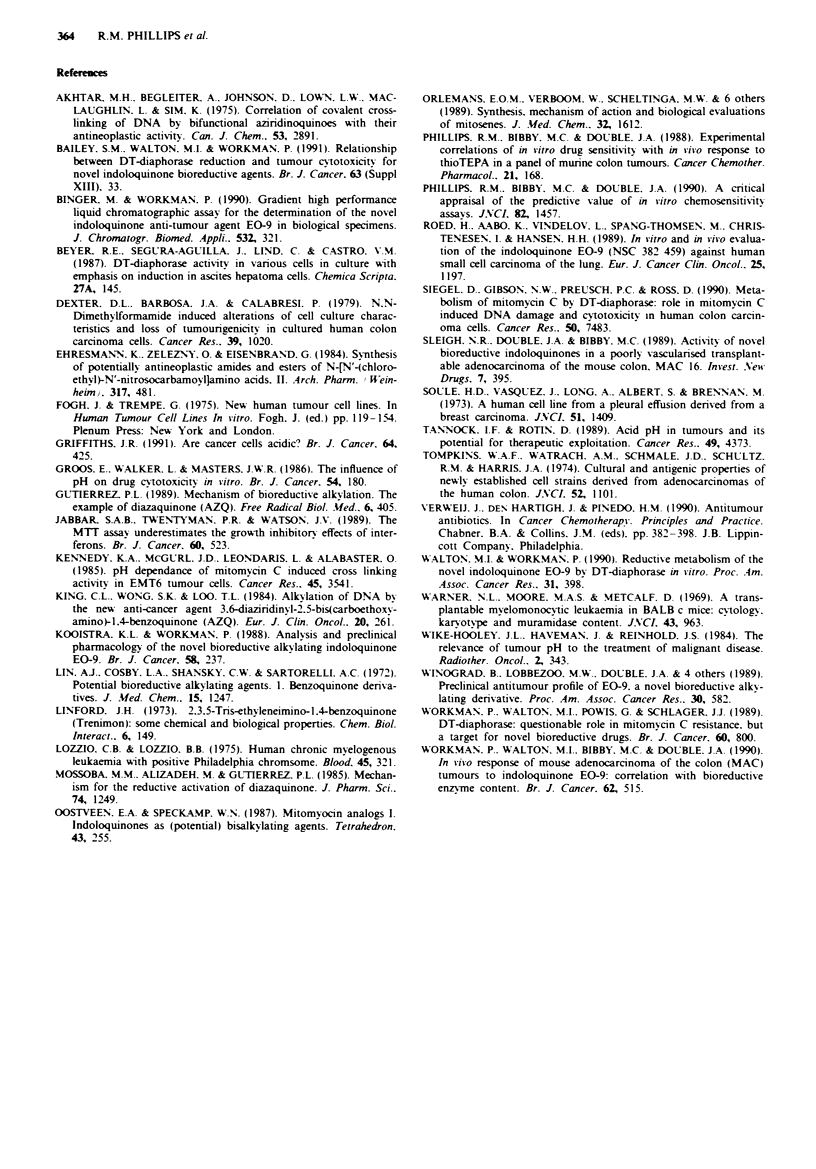# In vitro activity of the novel indoloquinone EO-9 and the influence of pH on cytotoxicity.

**DOI:** 10.1038/bjc.1992.73

**Published:** 1992-03

**Authors:** R. M. Phillips, P. B. Hulbert, M. C. Bibby, N. R. Sleigh, J. A. Double

**Affiliations:** Clinical Oncology Unit, School of Pharmacy, University of Bradford, West Yorkshire, UK.

## Abstract

The novel indoloquinone compound EO-9 is shortly to undergo phase I clinical evaluation as a potential bioreductive drug. Preclinical studies have shown that EO-9 has greater activity against cells derived from human solid tumours than leukaemias in vitro. The results of this study extend the preclinical data available on EO-9 by demonstrating that EO-9 induces a broad spectrum of activity (IC50 values range from 8 to 590 ng ml-1) against a panel of human and murine tumour cell lines. Some evidence exists of selectivity towards leukaemia and human colon cell lines as opposed to murine colon cells. The response of cells to Mitomycin C were not comparable to EO-9 suggesting that the mechanism of action of these compounds is different. The cytotoxic properties of EO-9 under aerobic conditions are significantly influenced by extracellular pH. Reduction of pH from 7.4 to 5.8 increases cell kill from 40% to 95% in DLD-1 cells. In addition, EO-9 is unstable at acidic pH (T1/2 = 37 min at pH 5.5) compared to neutral pH T1/2 = 6.3 h). The major breakdown product in vitro was identified as EO-5A which proved relatively inactive compared to EO-9 (IC50 = 50 and 0.6 ug ml-1 respectively). These studies suggest that if EO-9 can be delivered to regions of low pH within solid tumours, a therapeutic advantage may be obtained.


Br. J. Cancer (1992), 65, 359-364 © Macmillan Press Ltd., 1992

### *In vitro* activity of the novel indoloquinone EO-9 and the influence of pH on cytotoxicity

R.M. Phillips[1], P.B. Hulbert[2], M.C. Bibby[1], N.R. Sleigh[1] & J.A. Double[1]

[1]*Clinical Oncology Unit and* [2]*Pharmaceutical Chemistry, School of Pharmacy, University of Bradford, Bradford, West Yorkshire BD7 1DP, UK.*

**Summary** The novel indoloquinone compound EO-9 is shortly to undergo phase I clinical evaluation as a potential bioreductive drug. Preclinical studies have shown that EO-9 has greater activity against cells derived from human solid tumours than leukaemias *in vitro*. The results of this study extend the preclinical data available on EO-9 by demonstrating that EO-9 induces a broad spectrum of activity ($IC_{50}$ values range from 8 to 590 ng $ml^{-1}$) against a panel of human and murine tumour cell lines. Some evidence exists of selectivity towards leukaemia and human colon cell lines as opposed to murine colon cells. The response of cells to Mitomycin C were not comparable to EO-9 suggesting that the mechanism of action of these compounds is different. The cytotoxic properties of EO-9 under aerobic conditions are significantly influenced by extracellular pH. Reduction of pH from 7.4 to 5.8 increases cell kill from 40% to 95% in DLD-1 cells. In addition, EO-9 is unstable at acidic pH ($T_{\frac{1}{2}}$ = 37 min at pH 5.5) compared to neutral pH $T_{\frac{1}{2}}$ = 6.3 h). The major breakdown product *in vitro* was identified as EO-5A which proved relatively inactive compared to EO-9 ($IC_{50}$ = 50 and 0.6 ug $ml^{-1}$ respectively). These studies suggest that if EO-9 can be delivered to regions of low pH within solid tumours, a therapeutic advantage may be obtained.

In the search for new anti cancer drugs a series of novel indoloquinone compounds, based on the prototype bioreductive alkylating agent Mitomycin C (MMC), have been synthesised (Oostveen & Speckamp, 1987). The lead compound in this series, EO-9 [3-hydroxy-5-aziridinyl-1-methyl-2-(1H-indole-4, 7-dione)prop-β-en-α-ol] (Figure 1) has recently been selected for phase I clinical evaluation on the basis of good activity against human solid tumours over murine leukaemias *in vitro* and activity against several murine tumour models and human tumour xenografts *in vivo* (Winograd *et al.*, 1989). Bioreductive alkylation is considered to play an important role in the mechanism of action of EO-9 with the enzyme DT-diaphorase (NAD(P)H:(quinone acceptor) oxidoreductase (EC 1.6.99.2)) occupying a central role (Walton & Workman, 1990). It is believed that the two electron reduction of EO-9 via DT-diaphorase generates a reactive intermediate which can then undergo nucleophilic addition within biologically important molecules.

The concept of bioreductive activation stems from observations that hypoxic cells within a solid tumour mass exist in an environment that is more conducive to reductive reactions than their well oxygenated counterparts (Lin *et al.*, 1972). In addition to regions of hypoxia, solid tumours are known to possess several other properties which may influence the final outcome of chemotherapy. In particular, solid tumours are known to contain regions of low pH (Wike-Hooley *et al.*, 1984) generated as a result of a combination of poor blood supply, the production of lactic acid and the hydrolysis of ATP (Tannock & Rotin, 1989). The potential relevance of this established feature of solid tumour biology to determining anti-tumour responses is highlighted by several studies which demonstate that compounds containing the aziridine ring structure (e.g. ThioTEPA and several aziridinyl benzoquinones) are more potent in acidic rather than neutral pH conditions (Ahktar *et al.*, 1975; King *et al.*, 1984; Groos *et al.*, 1986; Phillips *et al.*, 1988). Similar studies have demonstrated that a reduction in extracellular pH potentiates MMC induced DNA cross links in EMT6 cells (Kennedy *et al.*, 1985). The aim of this study is 2-fold; firstly, to extend the preclinical data on EO-9 by comparing the response of a panel of human and murine tumour cell lines following exposure to EO-9 and MMC and secondly, to assess the influence of extracellular pH on the cytotoxic potency of EO-9 *in vitro*.

Correspondence: M.C. Bibby, Clinical Oncology Unit, University of Bradford, Bradford BD7 1DP, UK.
Received 19 August 1991; and in revised form 6 November 1991.

### Materials and methods

### *Cell lines and culture conditions*

A panel of human and murine tumour cell lines was employed, details of which are presented in Table I. All tumour cell lines, with the exception of K562 and WEHI-3B cells, were maintained as monolayer cultures in RPMI 1640 culture medium supplemented with 10% foetal calf serum, sodium pyruvate (1 mM), penicillin streptomycin (50 IU $ml^{-1}$ 50 μg $ml^{-1}$) and buffered by HEPES (25 mM). K562 and WEHI-3B cells were maintained as suspension cultures in RPMI 1640 as described above.

### *Drugs*

Formulated EO-9 was kindly provided by the New Drug Development Office of the EORTC. EO-9 was reconstituted in RPMI 1640 culture medium and stored at −20°C until required (no loss of cytotoxic activity was observed over a 2 month period despite repeated thawing and freezing). MMC was provided by the National Cancer Institute.

### *Chemosensitivity studies*

Cells were harvested from stock cultures in exponential growth and between 0.5 and 1 × $10^4$ viable cells in 180 μl of RPMI 1640 were plated into 96 well culture plates. Following an overnight incubation at 37°C, 20 μl of drug solution at an appropriate concentration were added to each well (8 wells per drug exposure) to yield a range of final EO-9 and MMC concentrations of 1 ng $ml^{-1}$ to 1 μg $ml^{-1}$. Following a 4 day incubation at 37°C in an atmosphere contaiing 5% $CO_2$ and

**Figure 1** The chemical structure of EO-9.

**Table I** The response of a panel of human and murine tumour cells following continuous exposure (96 h) to EO-9 and MMC

| Cell line | Cell line characteristics | EO-9 $IC_{50} \pm$ s.d. (ng ml$^{-1}$) | MMC $IC_{50} \pm$ s.d. (ng ml$^{-1}$) |
|---|---|---|---|
| MAC 15A | Murine ascitic tumour derived from a solid adenocarcinoma colon (Phillips *et al.*, 1990) | 430 ± 50 | 46 ± 17 |
| MAC 16 | Slow growing, cachectic murine adenocarcinoma colon (Phillips *et al.*, 1990) | 21 ± 9 | NA |
| MAC 26 | Well differentiated murine adenocarcinoma colon (Phillips *et al.*, 1990) | 590 ± 50 | 50 ± 15 |
| WEHI-3B | Murine myelomonocytic leukaemia (Warner *et al.*, 1969) | 95 ± 50 | 75 ± 23 |
| K562 | Human chronic myelogenous leukaemia (Lozzio & Lozzio, 1975) | 15 ± 4 | 76 ± 11 |
| HCLO | Human adenocarcinoma of the colon | 8 ± 3 | 55 ± 4 |
| HCT-18 | Human adenocarcinoma of the colon | 13 ± 3 | 183 ± 31 |
| HRT-18 | Human adenocarcinoma of the rectum (Tompkins *et al.*, 1974) | 8 ± 3 | 87 ± 48 |
| HT-29 | Human adenocarcinoma of the colon (Fogh & Trempe, 1975) | 18 ± 10 | 64 ± 21 |
| DLD-1 | Human adenocarcinoma of the colon (Dexter *et al.*, 1979) | 28 ± 20 | 133 ± 47 |
| MCF-7 | Derived from a pleural effusion of a human breast carcinoma (Soule *et al.*, 1973) | 17 ± 8 | 150 ± 32 |

All results presented are the means (± s.d.) of three independent experiments. NA = data not available.

95% air, chemosensitivity was assessed using the MTT assay (Jabbar *et al.*, 1989). Very briefly, 150 µl of old medium was removed and replaced with 150 µl of fresh medium immediately prior to the addition of 20 µl of MTT solution (5 mg ml$^{-1}$). Following a 4 h incubation at 37°C, 180 µl of medium plus MTT was removed from each well and the formazan crystals dissolved in 150 µl of DMSO. The absorbance of the resulting solution was read at 550 nm using an ELISA spectrophotometer. All results were expressed in terms of per cent survival taking the control absorbance values to represent 100% survival. Cytotoxic effects were expressed as $IC_{50}$ values (concentration required to reduce cell survival by 50%). All control cultures were in exponential growth at the time chemosensitivity was assessed.

*The influence of pH on the cytotoxic potency of EO-9*

MCF-7 and DLD-1 cell lines were considered to be the most appropriate cells to investigate the effect of pH on the cytotoxic properties of EO-9 principally because of their ability to form multicellular spheroids. As the response of spheroids and monolayers to EO-9 is the subject of future studies, an assessment of the influence of pH on EO-9 induced cell kill would have a bearing on the interpretation of these studies. MCF-7 and DLD-1 cell lines were harvested from stock cultures and $1 \times 10^4$ cells transferred to flat bottomed 96 well plates containing 200 µl of RPMI 1640 and incubated overnight at 37°C. Extracellular pH was altered by removing 195 µl of medium and replacing it with 180 µl of media at various pH values (pH was altered by adding small aliquots of 0.1 N NaOH or 0.1 N HCl to 10 ml of RPMI 1640). EO-9 (20 µl at the $IC_{50}$ value of 0.625 µg ml$^{-1}$) was added to each well and incubated at 37°C for 1 h. Following drug exposure cells were washed twice in Hanks balanced salt solution and chemosensitivity was assessed by the MTT assay described above. Control cultures at various pH were employed throughout. As an additional control experiment, the influence of pH on the cytotoxic properties of the novel nitrosourea, E10 (N-[N'-(2 chloroethyl)-N'-nitroso-carbamoyl]-L-alanine, Ehresmann *et al.*, 1984) were assessed using a 1 h exposure to 5 µg ml$^{-1}$. E10 is currently being evualated in this laboratory and was a gift from Prof. G. Eisenbrand, Kaiserslautern, Germany.

*Stability of EO-9 in RPMI 1640*

As the stability of MMC *in vitro* is strongly dependent upon pH (Verweji *et al.*, 1990) similar studies were conducted with EO-9. These studies in conjunction with the characterisation of the cytotoxic properties of the breakdown products *in vitro* would help to determine whether or not any increase in cytotoxic potency of EO-9 at low pH was due to increased reactivity of the compound itself or to the generation of a more cytotoxic breakdown product. Stability studies were conducted in RPMI 1640 culture medium at 37°C and at various pH values.

Chromatographic analysis of EO-9 in medium was based upon procedures published elsewhere (Kooistra & Workman, 1989). EO-9 was extracted from RPMI 1640 using C18 Bond Elut cartridge that had been primed with methanol (1 ml) and washed with distilled water (1 ml) prior to the addition of the sample (1 ml). Following a further washing step (1 ml distilled water), EO-9 was eluted in 200 µl methanol. The internal standard used was WV14 (Orlemans *et al.*, 1989). The extraction efficiency of EO-9 (1 µg ml$^{-1}$) and WV14 was 72 and 83% respectively. Extracted samples were injected into the HPLC and compounds were separated on a RP 18 column using an isocratic mobile phase of methanol $H_2O$ phosphate buffer (0.5 M) (45 54 1 % v v) at a flow rate of 1.2 ml min$^{-1}$. Compounds were detected at 280 nm (Waters spectrophotometer) and peak areas integrated using a Waters 740 data module. Drug concentrations were plotted against time. First order kinetics were assumed and log linear regression analysis used to determine the line of best fit. The half life was defined as the time taken for the drug concentration to decrease by 50%.

*Identification of breakdown products* in vitro

Breakdown products *in vitro* were generated by the addition of distilled water (2 ml) and a catalytic amount of acetic acid (50 µl of 5 M solution) to a solution of EO-9 (5.8 mg in 2 ml of acetonitrile). The reaction was allowed to proceed at 25°C for 3 h during which time aliquots (20 µl) were removed for spectroscopic monitoring of the reaction. The reaction solution, kept in a water bath at 40–45°C was evaporated to dryness under a stream of nitrogen gas, and the resulting solid freed from residual acetic acid by placing in a vacuum oven. Analysis of the product by mass spectrometry, UV spectroscopy and HPLC were conducted.

### Results

*Chemosensitivity* in vitro

The responses of a panel of human and murine tumour cell lines following continuous exposure to EO-9 are presented in Table I. A broad spectrum of activity exists ranging from the relatively resistant cell lines, MAC 15A and 26 ($IC_{50}$ values of 430 and 590 ng ml$^{-1}$ respectively) to sensitive cell lines particularly HCLO and HRT-18 ($IC_{50}$ = 8 ng ml$^{-1}$). Human colon cell lines (plus MCF-7) and the leukaemia cell lines tested were generally more responsive than the MAC cell lines with the exception of MAC 16 which is sensitive to EO-9 ($IC_{50}$ = 21 ng ml$^{-1}$). Furthermore, the spectrum of activity induced by EO-9 and MMC are not comparable (e.g. the cell line HCT-18 is very responsive to EO-9 whereas it is the most resistant line to MMC; Table I).

### *The influence of pH on the cytotoxic potency of EO-9*

The pH of RPMI 1640 was adjusted using aliquots of 0.1 N HCl determined from the calibration curve presented in Figure 2. Once adjusted, the pH of the medium remained stable over a 1 h period at 37°C (Figure 2). The responses of MCF-7 and DLD-1 cells exposed to EO-9 (1 h exposure to 0.625 $\mu g\ ml^{-1}$) in medium at different pH values are presented in Figure 3. Decreasing the extracellular pH below 7.5 significantly increases the cytotoxic potency of EO-9. Survival values at pH 7.4 and 5.8 were 60 and 5% respectively (Figure 3) in the case of DLD-1 cells and 55% and 7% for MCF-7 cells respectively. Increasing the pH above 7.5 had little or no effect on chemosensitivity. In the case of DLD-1 cells exposed to E10, decreasing the extracellular pH had no significant effect on cell survival with a 1 h exposure to 5 $\mu g\ ml^{-1}$ inducing cell kills of between 25 and 15% over a range of pH values (pH 5.5 to 8.0).

### *The stablility of EO-9* in vitro

The breakdown of EO-9 in RPMI 1640 medium at 37°C and at various pH values is presented in Figures 4 to 6. EO-9 is unstable under cell culture conditions with a half life of 6.3 h (Figure 4). One major breakdown product was observed which increased rapidly over the first 24 h before gradually plateauing (Figure 4). The breakdown of EO-9 significantly increased as the pH of the medium was reduced from 9.0 to 5.5 (Figure 5). The half life of EO-9 in RPMI 1640 medium at pH 9.0, 7.5, 6.0 and 5.5 was 12 h, 6.3 h, 2.5 h and 37 min respectively (Figure 5).

### *The identification of the breakdown product of EO-9 and its cytotoxic properties* in vitro

Chromatograms of EO-9 and its major breakdown product generated under acidic conditions are presented in Figures 6a and b respectively. Analysis of the breakdown product by HPLC showed that the product was a single compound (retention time 4.2 min) with less than 0.1% residual EO-9 remaining (retention time 5.3 min). Mass spectral data of EO-9 (Figure 7a) showed a molecular ion at 288.112 (100%)

**Figure 2** The volume of acid (0.1 N HCl) required to reduce the pH of RPMI 1640 medium (10 ml) at room temperature (O—O) and the pH of medium following a 1 h incubation at 37°C (◇—◇).

**Figure 3** The influence of pH on the cytotoxic potency of EO-9 (1 h exposure to 0.625 $\mu g\ ml^{-1}$) against MCF-7 (●—●) and DLD-1 (◆—◆) cells *in vitro*. The broken lines A, B and C denote the lowest recorded tumour pH, the mean tumour pH and the mean normal tissue pH respectively as observed by Wike-Hooley *et al.* (1984).

**Figure 4** The stability of EO-9 (●—●) in RPMI 1640 medium at 37°C (pH 7.4) and the generation of a major breakdown product (■—■). Concentration of the breakdown product is expressed in terms of EO-9 equivalents.

**Figure 5** The influence of pH on the stability of EO-9 in RPMI 1640 medium at 37°C; pH 9.0 (✱—✱), 7.5 (◆—◆), 6.0 (■—■) and 5.5 (●—●). Values in parenthesis denote half life.

**Figure 6** HPLC chromatograms of **a**. EO-9 in phosphate buffer at pH 7.5. **b**. EO-9 following a 3 h incubation in phosphate buffer (pH 4.5) at room temperature. Three breakdown products were detected. BP1 (the major breakdown product) and BP 2 and BP3 (minor break down products).

**Figure 7** Mass spectral data on **a**. EO-9 and **b**. its major breakdown product *in vitro*.

mass units by high resolution dynamic scanning ($C_{15}H_{16}N_2O_4$ requires 288.111) and fragments at 270 (16%), 257 (33%), 241 (51%), 229 (27%), 227 (28%), 213 (25%). The breakdown product generated following acid hydrolysis (5.1 mg, 16.7 μmoles, 83% yield) had a melting point of 176°C. Its mass spectrum (Figure 7b) showed a molecular ion at 306.126 (100%) mass units by high resolution dynamic scanning ($C_{15}H_{18}N_2O_5$ requires 306.122) and fragments at 290 (23%), 275 (50%), 257 (63%), 227 (41%).

Figure 8 shows the UV spectra of EO-9 in phosphate buffer at pH 7.5 and pH 4.6. The UV spectra of EO-9 at pH 7.5 remain stable over 30 min at room temperature (Figure 8a). Figure 8b shows the UV spectral changes in phosphate buffer at pH 4.6 that accompany the hydrolysis of EO-9 and the subsequent formation of the major breakdown product. EO-9 has a UV maximum at 271 nm (molar absorbance = 19,220) which shifts with increasing absorbance to 278 nm (24,020) (breakdown product). Similarly the peak at 312 nm (12,010 (EO-9)) shifts to 324 nm (11,860) (breakdown product). The breakdown product showed a new peak at 211 nm (17,770). Isobestic points are observed at 320 nm, 305 nm, 263 nm and 237 nm. These data are consistent with those of EO-5A (3-hydroxymethyl-5-(2-hydroxylethylamino)-1-methyl-2-(1H-indole-4,7-dione)-prop-β-en-α-ol) as described by Binger and Workman (1990).

In order to determine the relative cytotoxic properties of EO-9 and its major breakdown product, MAC 15A cells were continuously exposed to a range of drug and breakdown product concentrations and chemosensitivity assessed using the MTT assay. The results presented in Figure 9 demonstrate that the breakdown product is significantly less cytotoxic than EO-9 *in vitro* ($IC_{50}$ = 50 and 0.6 μg $ml^{-1}$ respectively).

### Discussion

The results presented in Table I clearly demonstrate that EO-9 is not only cytotoxic under standard cell culture conditions (5% $CO_2$, 95% air) but that it is capable of inducing a broad spectrum of activity in the panel of cell lines tested ($IC_{50}$ range = 8 to 590 ng $ml^{-1}$). The spectrum of activity

**Figure 8** **a**, UV spectra of EO-9 (20 μg $ml^{-1}$) in phosphate buffer at room temperature over a period of 30 mins (6 runs at 5 min intervals). **b**, The change in UV spectra of EO-9 (20 μg $ml^{-1}$) in phosphate buffer (pH 4.6) at room temperature over a period of 1 h (7 runs at time 0, 5, 10, 15, 20, 40 and 60 min).

**Figure 9** The response of MAC 15A cells to EO-9 (O—O) and the isolated breakdown product (●—●) *in vitro* (continuous exposure).

presented in this study is similar to that reported by Roed *et al.* (1989) although it differs from previous reports (Winograd *et al.*, 1989) in that no evidence of selectivity towards cells derived from human solid tumours over leukaemias exists in this study. Some evidence of selectivity towards leukaemia and human colon cell lines as opposed to MAC cell lines does however exist (Table I). Good responses *in vitro* to EO-9 have been shown to translate into good anti tumour activity *in vivo* (Roed *et al.*, 1989) and a similar correlation exists in the case of MAC 16 cells where both the cell line is sensitive to EO-9 ($IC_{50}$ = 21 ng $ml^{-1}$) and good anti tumour activity *in vivo* has been reported (Sleigh *et al.*, 1989). If this relationship holds true for other cell lines, then the possibility exists of conducting an '*in vitro* phase II' trial the aim of which would be to identify those tumour types that are most likely to respond to EO-9 in the clinic.

The reasons for the broad spectrum of activity observed are not known although the enzymology of the individual cell lines may be highly significant. Enzymes such as DT-diaphorase are believed to play a central role in the bioactivation of EO-9 (Walton & Workman, 1989) and a good correlation between the response of two MAC tumours and DT-diaphorase activity has been reported (Workman *et al.*, 1990). Similarly, the levels of DT-diaphorase in the HT-29 cell line are believed to be high (unpublished data in Workman *et al.*, 1990) which also correlates with good sensitivity to EO-9 in this study ($IC_{50}$ = 18 ng $ml^{-1}$). On the other hand, the good activity of EO-9 against the K562 cell line ($IC_{50}$ = 15 ng $ml^{-1}$) does not correlate with the fact that DT-diaphorase levels in this cell line are low (Beyer *et al.*, 1987). Further studies to characterise the activity of various enzymes within the panel of cell lines employed are currently in progress.

In terms of translating *in vitro* chemosensitivity data into anti tumour activity *in vivo*, numerous problems exist (Phillips *et al.*, 1990). This is particularly true in the case of bioreductive drugs where standard cell culture conditions do not mimic the complex tumour microenvironment against which these compounds are designed to act. Whilst drug exposure conditions *in vitro* can be modified to incorporate some of the features of solid tumour biology (such as low oxygen tension, pH etc), the fact that EO-9 is cytotoxic against cells under standard cell culture conditions suggests that well oxygenated cells within a solid tumour may also be a target. Provided sufficient quantities of EO-9 are delivered to a tumour for long enough, it is conceivable that any anti-tumour responses obtained may be due to a combination of direct cytotoxicity against aerobic cells as well as bioactivation in regions of hypoxia.

A comparison between the activity of EO-9 and MMC *in vitro* (Table I) demonstrates that major differences in the patterns of chemosensitivity exist suggesting that the mechanisms of action of these compounds is not the same. Studies in other laboratories have demonstrated that unlike EO-9, MMC is not a good substrate for DT-diaphorase (Workman *et al.*, 1989). It is also known that under aerobic conditions several enzyme systems are capable of activating MMC such as NADPH cytochrome P450 reductase, xanthine oxidase, some flavoprotein transhydrogenases (e.g. erythrocyte cytochrome $b_5$ reductase) as well as DT-diaphorase (Verweij *et al.*, 1990). Whilst the role of these enzymes in the activation of EO-9 are not fully understood, subtle differences in the substrate specificity of EO-9 and MMC may account for the different spectrum of activity observed.

Whilst differences between MMC and EO-9 exist, the two compounds are similar in that their stability and cytotoxic properties are pH dependent. Both EO-9 and MMC become more unstable (Figure 9 and Verweij *et al.*, 1990) and more active (Figure 3 and Kennedy *et al.*, 1985) as the pH is reduced. Two possible explanations may account for this property of EO-9. The first is that the metabolism of EO-9 by DT-diaphorase is enhanced under acidic conditions in a manner that is analogous to that of MMC (Seigel *et al.*, 1990) and secondly, that the chemical reactivity of the aziridine ring is enhanced under acidic conditions. In the case of MMC, Seigel *et al.* (1990) demonstrated that the metabolism of MMC by HT-29 cell cytosol (dicoumarol inhibitable) was pH dependent and increased as the pH was reduced to 5.8. For this mechanism to occur, it is essential that the intracellular pH decreases in line with a drop in extracellular pH. Kennedy *et al.* (1985) using flow cytometric analysis of the dye, 1,4 diacetoxy-2,3-dicyanobenzole, demonstrated that a decrease in extracellular pH is accompanied by a similar but smaller decrease in intracellular pH. On the other hand, measurements of intracellular pH by Magnetic Resonance Spectroscopy have shown that the intracellular pH of cells within tumours is in fact neutral or slightly alkaline (Griffiths, 1991). In this case it is doubtful that an increase in the metabolism of EO-9 by DT-diaphorase within the cell will explain the observations presented in this study.

Alternatively, the chemical reactivity of EO-9 may be enhanced at low pH values. It is known for instance that the reactivity of aziridine groups is facilitated by protonation resulting in the opening of the aziridine ring to release ring strain energy (Mossoba *et al.*, 1985). This generates an aziridinium ion which is a potent alkylating species (Lindford, 1973; Gutierrez, 1989). Increased reactivity of the aziridine ring has been proposed to explain the increased DNA cross linking activity of MMC and aziridinyl benzoquinones (Akhtar *et al.*, 1975; Kennedy *et al.*, 1985). Facilitation of aziridine ring opening may similarly explain the increased cytotoxic potency of EO-9 in acidic conditions particularly as the major breakdown product of EO-9 *in vitro* (EO-5A) is relatively inactive (Figure 9 and Bailey *et al.*, 1991). Finally, the fact that the activity of E10 is not influenced by low pH together with similar reports using doxorubicin, epirubicin and epodyl (Groos *et al.*, 1986) suggest that the subjection of tumour cells to low pH stress does not in itself make the cells more sensitive to any cytotoxic insult.

The therapeutic implications of these results are not known, particularly in view of the controversy concerning the pH of malignant tissues (Wike-Hooley *et al.*, 1984; Griffiths, 1991). Nevertheless, a review of microelectrode measurements of normal and malignant tissue pH (extracellular) demonstrates that the pH of tumour tissue is lower than that of normal subcutis or muscle tissue (Wike-Hooley *et al.*, 1984). When these figures are superimposed upon Figure 3, the cell kill induced at the lowest recorded pH of 5.8 is significantly greater than that at the mean pH of normal tissues which suggests in these cases that a therapeutic advantage may be obtained.

In conclusion, the results of this study demonstrate that major differences in the inherent chemosensitivity of individual cell lines to EO-9 do exist. Further studies are required to determine whether or not the responses observed *in vitro* translate into antitumour activity *in vivo* and to correlate enzyme activity in each cell line with cytotoxic effects *in vitro*. If the correlation is strong, then the targetting of EO-9 against particular tumour types in the clinic may become a viable proposition. The demonstration that the cytotoxic properties of EO-9 are influenced by extracellular pH *in vitro* introduces another variable factor that may influence the final outcome of chemotherapy *in vivo*.

This work was supported by Bradfords War on Cancer and the International Association for Cancer Research. The authors wish to thank Mr R.A. Powell for technical assistance in conducting the influence of pH on cytotoxicity.

### References

AKHTAR, M.H., BEGLEITER, A., JOHNSON, D., LOWN, L.W., MACLAUGHLIN, L. & SIM, K. (1975). Correlation of covalent cross-linking of DNA by bifunctional aziridinoquinoes with their antineoplastic activity. *Can. J. Chem.*, **53**, 2891.

BAILEY, S.M., WALTON, M.I. & WORKMAN, P. (1991). Relationship between DT-diaphorase reduction and tumour cytotoxicity for novel indoloquinone bioreductive agents. *Br. J. Cancer*, **63** (Suppl XIII), 33.

BINGER, M. & WORKMAN, P. (1990). Gradient high performance liquid chromatographic assay for the determination of the novel indoloquinone anti-tumour agent EO-9 in biological specimens. *J. Chromatogr. Biomed. Appli.*, **532**, 321.

BEYER, R.E., SEGURA-AGUILLA, J., LIND, C. & CASTRO, V.M. (1987). DT-diaphorase activity in various cells in culture with emphasis on induction in ascites hepatoma cells. *Chemica Scripta*, **27A**, 145.

DEXTER, D.L., BARBOSA, J.A. & CALABRESI, P. (1979). N,N-Dimethylformamide induced alterations of cell culture characteristics and loss of tumourigenicity in cultured human colon carcinoma cells. *Cancer Res.*, **39**, 1020.

EHRESMANN, K., ZELEZNY, O. & EISENBRAND, G. (1984). Synthesis of potentially antineoplastic amides and esters of N-[N'-(chloroethyl)-N'-nitrosocarbamoyl]amino acids, II. *Arch. Pharm. (Weinheim)*, **317**, 481.

FOGH, J. & TREMPE, G. (1975). New human tumour cell lines. In *Human Tumour Cell Lines In vitro*. Fogh, J. (ed.) pp. 119-154. Plenum Press: New York and London.

GRIFFITHS, J.R. (1991). Are cancer cells acidic? *Br. J. Cancer*, **64**, 425.

GROOS, E., WALKER, L. & MASTERS, J.W.R. (1986). The influence of pH on drug cytotoxicity *in vitro*. *Br. J. Cancer*, **54**, 180.

GUTIERREZ, P.L. (1989). Mechanism of bioreductive alkylation. The example of diazaquinone (AZQ). *Free Radical Biol. Med.*, **6**, 405.

JABBAR, S.A.B., TWENTYMAN, P.R. & WATSON, J.V. (1989). The MTT assay underestimates the growth inhibitory effects of interferons. *Br. J. Cancer*, **60**, 523.

KENNEDY, K.A., MCGURL, J.D., LEONDARIS, L. & ALABASTER, O. (1985). pH dependance of mitomycin C induced cross linking activity in EMT6 tumour cells. *Cancer Res.*, **45**, 3541.

KING, C.L., WONG, S.K. & LOO, T.L. (1984). Alkylation of DNA by the new anti-cancer agent 3,6-diaziridinyl-2,5-bis(carboethoxyamino)-1,4-benzoquinone (AZQ). *Eur. J. Clin. Oncol.*, **20**, 261.

KOOISTRA, K.L. & WORKMAN, P. (1988). Analysis and preclinical pharmacology of the novel bioreductive alkylating indoloquinone EO-9. *Br. J. Cancer*, **58**, 237.

LIN, A.J., COSBY, L.A., SHANSKY, C.W. & SARTORELLI, A.C. (1972). Potential bioreductive alkylating agents. 1. Benzoquinone derivatives. *J. Med. Chem.*, **15**, 1247.

LINFORD, J.H. (1973). 2,3,5-Tris-ethyleneimino-1,4-benzoquinone (Trenimon): some chemical and biological properties. *Chem. Biol. Interact.*, **6**, 149.

LOZZIO, C.B. & LOZZIO, B.B. (1975). Human chronic myelogenous leukaemia with positive Philadelphia chromsome. *Blood*, **45**, 321.

MOSSOBA, M.M., ALIZADEH, M. & GUTIERREZ, P.L. (1985). Mechanism for the reductive activation of diazaquinone. *J. Pharm. Sci.*, **74**, 1249.

OOSTVEEN, E.A. & SPECKAMP, W.N. (1987). Mitomyocin analogs I. Indoloquinones as (potential) bisalkylating agents. *Tetrahedron*, **43**, 255.

ORLEMANS, E.O.M., VERBOOM, W., SCHELTINGA, M.W. & 6 others (1989). Synthesis, mechanism of action and biological evaluations of mitosenes. *J. Med. Chem.*, **32**, 1612.

PHILLIPS, R.M., BIBBY, M.C. & DOUBLE, J.A. (1988). Experimental correlations of *in vitro* drug sensitivity with *in vivo* response to thioTEPA in a panel of murine colon tumours. *Cancer Chemother. Pharmacol.*, **21**, 168.

PHILLIPS, R.M., BIBBY, M.C. & DOUBLE, J.A. (1990). A critical appraisal of the predictive value of *in vitro* chemosensitivity assays. *JNCI*, **82**, 1457.

ROED, H., AABO, K., VINDELOV, L., SPANG-THOMSEN, M., CHRISTENESEN, I. & HANSEN, H.H. (1989). *In vitro* and *in vivo* evaluation of the indoloquinone EO-9 (NSC 382 459) against human small cell carcinoma of the lung. *Eur. J. Cancer Clin. Oncol.*, **25**, 1197.

SIEGEL, D., GIBSON, N.W., PREUSCH, P.C. & ROSS, D. (1990). Metabolism of mitomycin C by DT-diaphorase: role in mitomycin C induced DNA damage and cytotoxicity in human colon carcinoma cells. *Cancer Res.*, **50**, 7483.

SLEIGH, N.R., DOUBLE, J.A. & BIBBY, M.C. (1989). Activity of novel bioreductive indoloquinones in a poorly vascularised transplantable adenocarcinoma of the mouse colon, MAC 16. *Invest. New Drugs*, **7**, 395.

SOULE, H.D., VASQUEZ, J., LONG, A., ALBERT, S. & BRENNAN, M. (1973). A human cell line from a pleural effusion derived from a breast carcinoma. *JNCI*, **51**, 1409.

TANNOCK, I.F. & ROTIN, D. (1989). Acid pH in tumours and its potential for therapeutic exploitation. *Cancer Res.*, **49**, 4373.

TOMPKINS, W.A.F., WATRACH, A.M., SCHMALE, J.D., SCHULTZ, R.M. & HARRIS, J.A. (1974). Cultural and antigenic properties of newly established cell strains derived from adenocarcinomas of the human colon. *JNCI*, **52**, 1101.

VERWEIJ, J., DEN HARTIGH, J. & PINEDO, H.M. (1990). Antitumour antibiotics. In *Cancer Chemotherapy. Principles and Practice*. Chabner, B.A. & Collins, J.M. (eds). pp. 382-398. J.B. Lippincott Company, Philadelphia.

WALTON, M.I. & WORKMAN, P. (1990). Reductive metabolism of the novel indoloquinone EO-9 by DT-diaphorase *in vitro*. *Proc. Am. Assoc. Cancer Res.*, **31**, 398.

WARNER, N.L., MOORE, M.A.S. & METCALF, D. (1969). A transplantable myelomonocytic leukaemia in BALB c mice: cytology, karyotype and muramidase content. *JNCI*, **43**, 963.

WIKE-HOOLEY, J.L., HAVEMAN, J. & REINHOLD, J.S. (1984). The relevance of tumour pH to the treatment of malignant disease. *Radiother. Oncol.*, **2**, 343.

WINOGRAD, B., LOBBEZOO, M.W., DOUBLE, J.A. & 4 others (1989). Preclinical antitumour profile of EO-9, a novel bioreductive alkylating derivative. *Proc. Am. Assoc. Cancer Res.*, **30**, 582.

WORKMAN, P., WALTON, M.I., POWIS, G. & SCHLAGER, J.J. (1989). DT-diaphorase: questionable role in mitomycin C resistance, but a target for novel bioreductive drugs. *Br. J. Cancer*, **60**, 800.

WORKMAN, P., WALTON, M.I., BIBBY, M.C. & DOUBLE, J.A. (1990). *In vivo* response of mouse adenocarcinoma of the colon (MAC) tumours to indoloquinone EO-9: correlation with bioreductive enzyme content. *Br. J. Cancer*, **62**, 515.